# Loss of Progesterone Receptor-Mediated Actions Induce Preterm Cellular and Structural Remodeling of the Cervix and Premature Birth

**DOI:** 10.1371/journal.pone.0081340

**Published:** 2013-12-10

**Authors:** Steven M. Yellon, Abigail E. Dobyns, Hailey L. Beck, James T. Kurtzman, Robert E. Garfield, Michael A. Kirby

**Affiliations:** 1 Division of Physiology, Department of Basic Sciences and Center for Perinatal Biology, Loma Linda University School of Medicine, Loma Linda, California, United States of America; 2 Department of Pediatrics, Pathology and Human Anatomy, Loma Linda University School of Medicine, Loma Linda, California, United States of America; 3 Department of Obstetrics and Gynecology, Loma Linda University School of Medicine, Loma Linda, California, United States of America; 4 St. Joseph's Hospital and Medical Center, Department of Obstetrics and Gynecology, Phoenix, Arizona, United States of America; Queen's University, Canada

## Abstract

A decline in serum progesterone or antagonism of progesterone receptor function results in preterm labor and birth. Whether characteristics of premature remodeling of the cervix after antiprogestins or ovariectomy are similar to that at term was the focus of the present study. Groups of pregnant rats were treated with vehicle, a progesterone receptor antagonist (onapristone or mifepristone), or ovariectomized on day 17 postbreeding. As expected, controls given vehicle delivered at term while rats delivered preterm after progesterone receptor antagonist treatment or ovariectomy. Similar to the cervix before term, the preterm cervix of progesterone receptor antagonist-treated rats was characterized by reduced cell nuclei density, decreased collagen content and structure, as well as a greater presence of macrophages per unit area. Thus, loss of nuclear progesterone receptor-mediated actions promoted structural remodeling of the cervix, increased census of resident macrophages, and preterm birth much like that found in the cervix at term. In contrast to the progesterone receptor antagonist-induced advance in characteristics associated with remodeling, ovariectomy-induced loss of systemic progesterone did not affect hypertrophy, extracellular collagen, or macrophage numbers in the cervix. Thus, the structure and macrophage census in the cervix appear sufficient for premature ripening and birth to occur well before term. With progesterone receptors predominantly localized on cells other than macrophages, the findings suggest that interactions between cells may facilitate the loss of progesterone receptor-mediated actions as part of a final common mechanism that remodels the cervix in certain etiologies of preterm and with parturition at term.

## Introduction

The cervix is a critical gatekeeper for parturition. At term across a variety of mammalian species, structural changes that resemble an inflammatory process occur in the cervix well before onset of active labor [1]–[4]. Findings in the prepartum cervix of several strains and species of rodents at term indicate reduced density of cells in the subepithelium and stroma, degradation of extracellular collagen matrix, and increased numbers of macrophages/area relative to earlier pregnancy or in nonpregnant controls [3]–[6]. More macrophages in the prepartum cervix of mice were recently confirmed by flow cytometry [6]. These characteristics associated with cervical remodeling occur near the peak of serum progesterone concentrations, 2–4 days before the onset of labor at term in rodents [7]–[10]. Moreover post-peak progesterone in circulation during the last days of pregnancy are significantly greater than concentrations during the estrous cycle peak in nonpregnant females [11]–[15] and actually exceed levels reported to saturate tissue progesterone receptors [16], [17]. Thus during the process of cervical remodeling, sustained systemic progesterone in mice and rats resembles that in women where serum progesterone does not decline until shortly after birth.

The importance of a more local loss of the trophic actions of progesterone as part of the final common pathway for remodeling the cervix and parturition is well supported in a variety of species. Antagonists to the progesterone receptor are known to terminate pregnancy in women [18]–[20] and induces preterm birth in rodents [21]–[23]. Antiprogestin-induced remodeling of the cervix is associated with increased collagenase activity, decrease collagen concentrations, apoptosis, cell proliferation, degradation of the extracellular collagen matrix, as well as decreased cervical resistance to stretch and other biomechanical properties typical of the prepartum cervix by term [24]. With antiprogestin treatment, an increased prevalence of leukocytes in the uterine cervix has been reported [5], [25]. As the predominant leukocyte, assessments of macrophages in the cervix are complicated by a heterogeneous and high variability distribution of immune cells within subepithelial and stromal subregions, as well as, in the transition zone between the Os and uterine horns [4], [6]. Thus to accurately evaluate the census of macrophages, a survey of microscopic fields needs to take into account the increased cellular hypertrophy over diverse subregions of the cervix with progress to term. By example, a report of mifepristone (RU486)-induced increased prevalence of macrophages in the cervix in anticipation of preterm birth was based upon evidence of increased non-cellular staining in mucus and epithelium, but not evaluation of the census of macrophages within stroma [26]. In addition, experiences with collagen stain indicate that dye intensity may vary with respect to i) comparability and quality of sections, ii) processing of slides on different days, as well as iii) illumination settings. As such, critical features of the remodeling process after loss of progestational support have yet to be investigated in the prepartum cervix with preterm birth.

In the present study, the first goal was to evaluate whether remodeling characteristics of the cervix at term are advanced by antagonism of classic nuclear progesterone receptors. The second objective of this investigation was based upon well-established evidence that removal of the ovaries in rats results in preterm birth [27], [28]. The novel investigation of cellular and structural remodeling of the cervix that follows premature loss of systemic progesterone tested the hypothesis that critical characteristics of a final common mechanism for cervical remodeling are similarly regulated as in preterm loss of the trophic actions of progesterone, as well as at term. Our findings suggest that preterm birth induced by blockade of progesterone receptors, but not systemic loss of progesterone, advances structural and cellular changes in the peripartum cervix comparable to the remodeling process associated with birth at term. In addition, evidence is provided to indicate that macrophages in the peripartum cervix may not directly mediate the effects of progesterone receptor blockade-induced preterm remodeling.

## Materials and Methods

Animal care followed guidelines set by the National Institute of Health and experimental procedures were approved by the Loma Linda University Institutional Animal Care and Use Committee. Time-dated gravid 10 week old female Sprague-Dawley rats were purchased from Harlan (Livermore, CA). Rats were housed with free access to food and water in a humidity-controlled vivarium room at 23°C with lights on for 12 h (7am –7pm PDT). During the study period, rats were observed at about 2 h intervals between 9 am and 5pm. The presence of pups or tissue by 9 am indicated that birth had occurred within the previous 14 h.

On the morning of day 17 post-breeding (approximately 75% of gestation), pregnant rats were randomly assigned to one of four groups and injected with either 1) vehicle (Con; 0.1 ml s.c. of 1∶7 ethanol: sesame oil), or 2) Onapristone (Ona, a classic nuclear progesterone receptor antagonist; 10 mg/kg body weight/0.1 ml vehicle s.c.[29], or 3) RU486 (a mixed nuclear progesterone and glucocorticoid receptor antagonist; 10 mg/kg body weight/0.1 ml vehicle s.c.; Sigma-Aldrich, St Louis, MO), or 4) bilaterally ovariectomized (Ovx) under 4% fluorothane gas anesthesia. Rats in each group (n = 4–7/postbreeding day) were asphyxiated with CO_2_ about 8 h postsurgery (D17.5), the next morning about 24 h post-treatment or surgery (D18), or postpartum on the morning of delivery (PP). In addition, a group of Con rats were euthanized in the afternoon of the day preceding expected birth (D21.5). Immediately postmortem, a blood sample was obtained by cardiac puncture and, after overnight at 4C, serum was obtained then frozen at −20C for later assay of progesterone (described below). The cervix was removed from each rat, post-fixed overnight, embedded in paraffin, sectioned longitudinally at 10 μm, and processed to assess collagen in the extracellular matrix or cell nuclei and resident macrophages as previously described [6].

### Staining and analyses of cervix sections

Sections were processed by immunohistochemistry to stain macrophages (ED-1 antigen = CD68) and counterstained with hematoxylin or methyl green to identify cell nuclei. As a well-characterized marker of differentiated macrophages, ED-1 antibody-stained cells in the cervix were counted in a calibrated field of view in brightfield photomicrographs of cervical sections from each rat as before [6], [30]. Other immune cells that may cross-react with ED-1 are rarely present in the cervix [31]. ED-1 stained cells appeared to contain dark brown deposits, ranging from sparse patches to a completely filled cell body (HRP-DAB, horseradish peroxidase-diaminobenzidine deposits). For a cell to be counted as a macrophage, this stain was required to be associated with a single counterstained cell nucleus, distinct from the border of the photomicrograph. Typically, photomicrographs of stroma from the Os to striated border with the uterine body were captured without blood vessel or epithelium adjacent to the lumen. As in previous studies, random representative photomicrographs (40x lens) in sections from each cervix were reviewed by multiple, trained personnel to assure an accurate enumeration of cell nuclei and immunostained cells [3]. Briefly, the number of counterstained cell nuclei and immunostained cells were counted in 6 sections/rat, 6–8 photomicrographs of non-overlapping regions per section, i.e. analysis of 36–48 photomicrographs (average of 930,025 μm^3^/rat, empirically determined to have a coefficient of variance <15%). The average number of immunostained cells/area of photomicrograph was divided by the average cell nuclei/area (cell density) for each individual to account for cellular hypertrophy or edema within the same area of tissue used to enumerate macrophages.

Sections of cervix from prepartum and postpartum rats in Con groups (D17.5, 18, and 22 postbreeding =  day of term birth), as well as prepartum and postpartum groups of rats that deliver preterm after Ona or RU486 or Ovx (n = 3–4/group at each postbreeding day) were processed by immunohistochemistry to identify ED-1 stained macrophages (HRP-DAB) that also have progesterone receptors (rabbit anti-progesterone receptor A/B, Cell signaling technologies, Danvers, MA, Alkaline phosphatase-Vector Red). Based upon previous procedures [30], [32], antigen retrieval of duplicate10 μm sections was followed by an overnight incubation at 4C with the first primary antibody (ED-1, 1∶50 dilution, Serotec, Raleigh, NC), visualization the next day (anti-Mouse IgG 1∶200 dilution and ABC, Vector Labs, Burlingame, CA), a second overnight incubation with the second primary antibody (PR AB, 1∶200 dilution, Cell Signaling Technology, Danvers, MA), and visualization the next day (anti-Rabbit IgG 1∶200 dilution, Invitrogen, Grand Island, NY; Vectastain, streptavidin, Sigma Aldrich, St. Louis, MO; Vector Red, Vector Labs). Sections were washed, dehydrated, and coverslipped.

Collagen content and structure was assessed in cervix sections after picrosirius red staining as previously described [2]–[4], [30]. This assessment of extracellular collagen is in the actual anatomical context in which macrophages reside and has been found to correlate with progression of the remodeling process in multiple studies in two species using two different microscope systems. Briefly, a black-and-white photomicrograph was taken from stromal and subepithelial subregions of cervix (exclusive of epithelium and blood vessels) with a 20x objective and controlled light settings to assess birefringence of polarized transmitted light. Nine adjacent, but non-overlapping photomicrographs were taken in each of 3 sections/rat (total of 27 images/cervix  = 675,000 µm^3^). The optical density of these images was then quantified using the Rodbard grayscale threshold calibration (NIH Image), calculation that yields an optical density measure that is inversely related to extracellular collagen content and structure. Specifically, transmitted light from bright picrosirius red stained areas, indicative of high collagen content and complex cross-linking, produce low optical density numbers. However, dim regions with low light transmittance had high optical density values – indicative of minimal staining and low collagen content, as well as reduced cross-linking. Optical density data were normalized with respect to average cell nuclei density (defined above) for each individual cervix section to control for possible local variations in hypertrophy or edema of tissue. Thus, as in previous studies in several strains of rat and mice, this approach facilitated evaluation of extracellular collagen content and structural organization of fibers in the stroma and perimetrium subregions of the cervix and in comparison with various treatment groups.

### Progesterone assay and statistical analyses

Serum progesterone was assayed in duplicate (Cat#582601, Cayman Chemical Co, Ann Arbor, MI). Assay sensitivity was <0.2 ng/ml, while inter- and intra-assay variability were <14.7%. All data were normally distributed (Levene's test p>0.05). Data within groups or across days of pregnancy among groups were evaluated by one-way ANOVA followed by Tukey's post-hoc test for individual comparisons (GraphPad Prism, La Jolla, CA). This parametric analysis and stringency in multiple comparisons permitted evaluation of data among groups relative to time after treatment, as well as time before birth regardless of day of pregnancy. p<0.05 was considered significant (collectively, F>5.2, df range from 13–21). The Student's t-test was used to compare data for the two Ona groups and other comparisons as specified in Figure legends. Data are reported as the mean ± SEM.

## Results

### Time of birth and peripartum changes in progesterone in circulation

Rats in the control group delivered on average on day 22 as expected for a normal term delivery for this strain. In contrast, all rats administered Ona gave birth preterm by noon on day 18 post breeding (Ona PP), within 27 h of treatment. RU486-treated rats also delivered preterm; 6 of the 11 births occurred by 1pm on day 18 postbreeding (average of 26 h after treatment). For Ovx rats, removal of the ovaries in the morning of day 17 postbreeding led to delivery of pups by the morning of day 19 (Ovx PP, n = 5), about 14 h later than that in PR antagonist-treated rats (36 h-44 h after surgery) and two days early compared to controls. In 2 of 5 postpartum Ovx rats, some pups were found with bruising and delivery was not complete since at least one pup remained *in utero*. For these rats, an accurate estimate for the duration of labor could not be determined.

**Figure 1:**
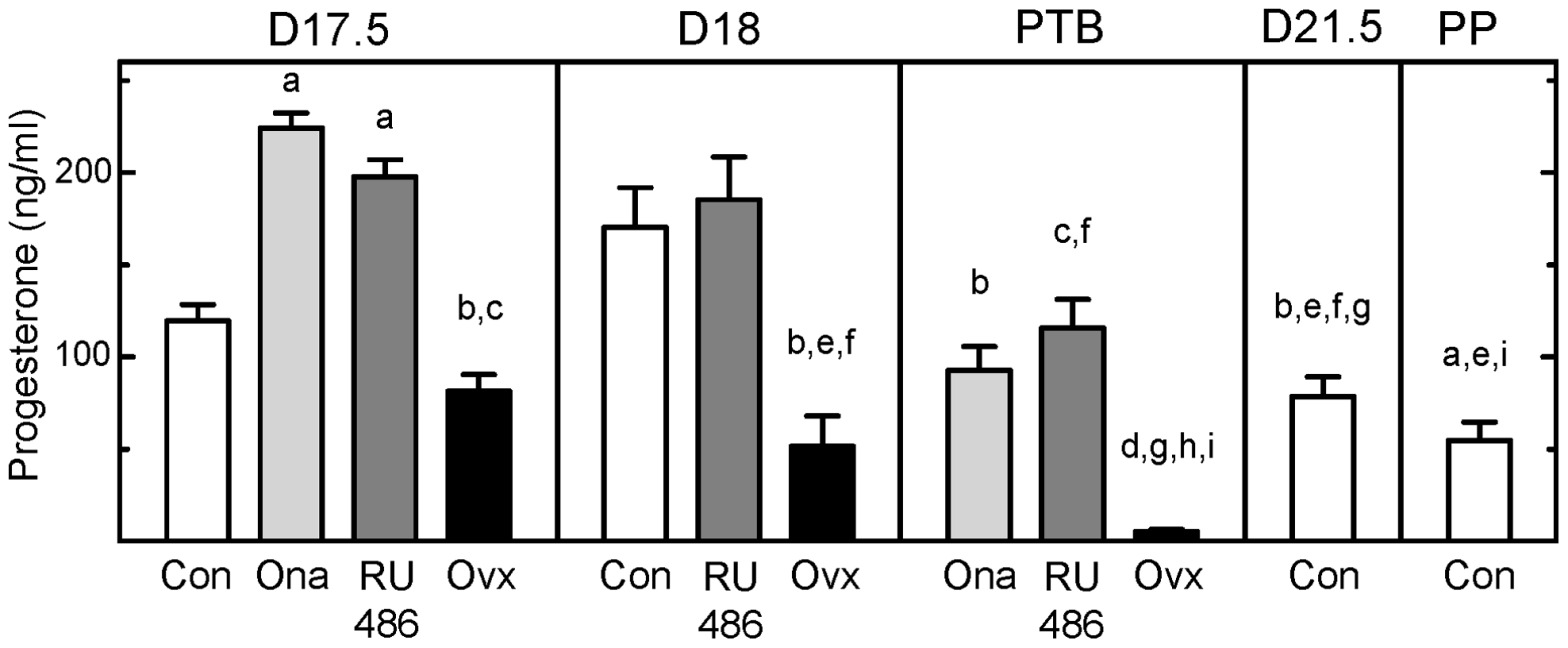
Peripartum progesterone in circulation in preterm and term birth. Serum progesterone concentrations (mean±SE, n = 4–6/group) on various days postbreeding in controls, and groups of pregnant rats treated with Onapristone (Ona) or RU486 or bilaterally ovariectomized on the morning of day 17 of pregnancy (Ovx). Preterm birth (PTB) occurred on day 18 postbreeding for Ona- and RU486-treated rats, by 27 h or 26 h post-treatment, respectively (n = 5 and 6 respectively). PTB occurred by the morning of day 19 postbreeding in the Ovx group (36–44 h post-surgery, n = 5). Postpartum (PP) is the morning of the day of birth at term on day 22 postbreeding in Cons (n = 5). Typical estrous cycle serum progesterone concentrations range from 5–50 ng/ml in nonpregnant rats [12]. For statistical comparisons, groups were assigned an individual letter, consecutively from ^a^ for Con D17.5 to ^l^ for Con PP groups. The letter above a bar indicates p<0.05 vs the respective treatment group (ANOVA with Tukey's test for individual comparisons). By example, ^a^ over bars for Ona D17.5, RU486 D17.5 and Con PP groups indicates a significant difference for each versus the Con D17.5 group. The absence of a letter, ^k^ for example, indicates no statistical difference for any group compared to Con D21.5. See Methods for experimental details.

Progesterone concentrations in controls were similar to values previously reported during pregnancy and the peripartum period [12], [13]. By the day before birth, a significant decline in serum progesterone was evident in controls (Figure 1, Con D21.5 vs D18). In contrast, progesterone concentrations in PR antagonist-treated pregnant rats were higher in Ona- and RU486-treated rats compared to that in controls on days 17.5 postbreeding or the prepartum D21.5 group at term. Relative to the prepartum peak, postpartum progesterone in circulation declined after preterm birth in rats given either antagonist; concentrations remained elevated versus those in controls on the day of birth and significantly so for RU486-treated rats. In Ovx rats, serum progesterone decreased within 24 h of surgery relative to that in controls on day 18 postbreeding and declined further by day 19 postbreeding when pups delivered preterm. Serum progesterone was lower in Ovx groups compared to that in Ona- and RU486-treated rats on the same day postbreeding.

### Morphology of cervix with preterm birth

**Figure 2:**
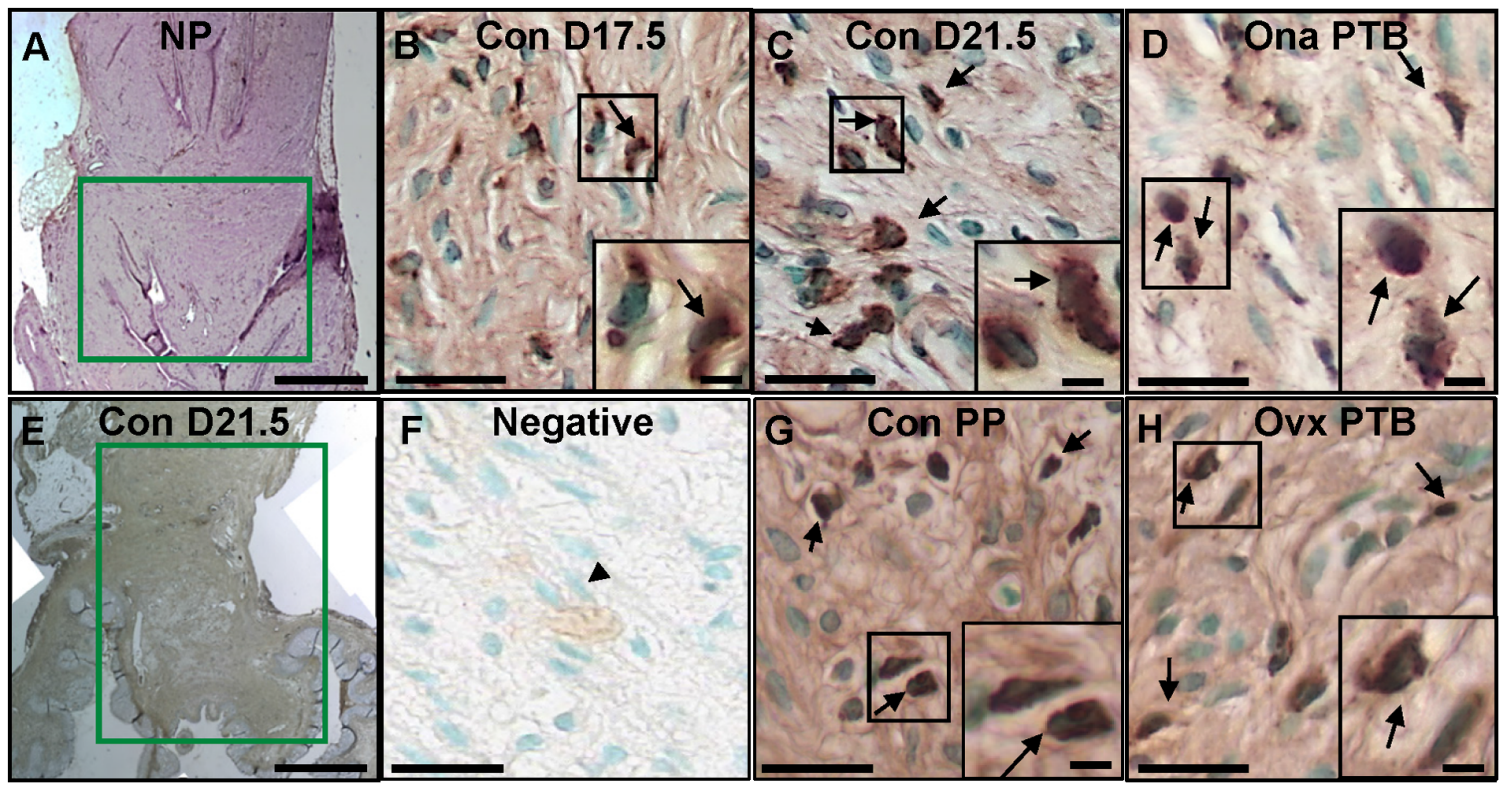
Photomicrographs of rat cervix sections for comparison of gross and cellular anatomy. Stitched multiple photomicrographs of a cervix section stained with the pan-macrophage marker ED-1 from **A**. a nonpregnant (NP, hematoxylin counterstain [30]) and **E**. a prepartum control rat (Con D21.5, day 21.5 postbreeding, methyl green counterstain). Green box highlights cervix lengthening and hypertrophy with pregnancy (scale bar = 1 mm). Vaginal folds surround the Os while uterine tissue body is generally above the green box, though a clear border between the cervix and uterus is difficult to define. Other photomicrographs of ED-1-stained, methyl green counterstained cervix sections from control rats on day 17.5 or 21.5 postbreeding or postpartum day 22 postbreeding (**B**. Con D17.5, **C**. Con D21.5, or **G**. Con PP, respectively) or from a rat treated with **D**. Ona or **H**. ovariectomized (Ovx) on day 17 postbreeding with preterm birth (PTB). **F**. Negative is a section from a Con D21.5 cervix that was processed without primary ED1 antibody (nonspecific background is not associated with methyl green-stained cell nucleus – example specified with black arrowhead). Box indicates area magnified in insets; examples of fully-stained macrophages with brown deposits are shown by arrows. Photomicrographs were taken at 40x and sized to fit; scale bars in 4 right panels are 25 μm and 5 μm for insets, respectively.

In a macroscopic view, the cervix extends from the Os which protrudes into the vagina surrounded by vaginal folds (Figure 2 A, E). At the confluence of the cervix and uterus, layers of smooth muscle and glands are evident in a transition zone with horizontal striations that densely stain for collagen. In comparison to nonpregnant rats from a previous study [30], the length of the prepartum cervix between the Os and the transitional striated region proximal to the uterus increased (note scale bar difference). With pregnancy, cervical cells appeared increased in size and more blood vessels with greater internal volume were present in the stroma versus that in nonpregnant rats. Macrophages were clearly identified by dark brown stain that surrounded a methyl green counterstained cell nucleus in sections from the cervix of Con groups (days 17.5 and 21.5 postbreeding) and preterm postpartum Ona and Ovx rats (Figure 2 B–D, G, H).

**Figure 3:**
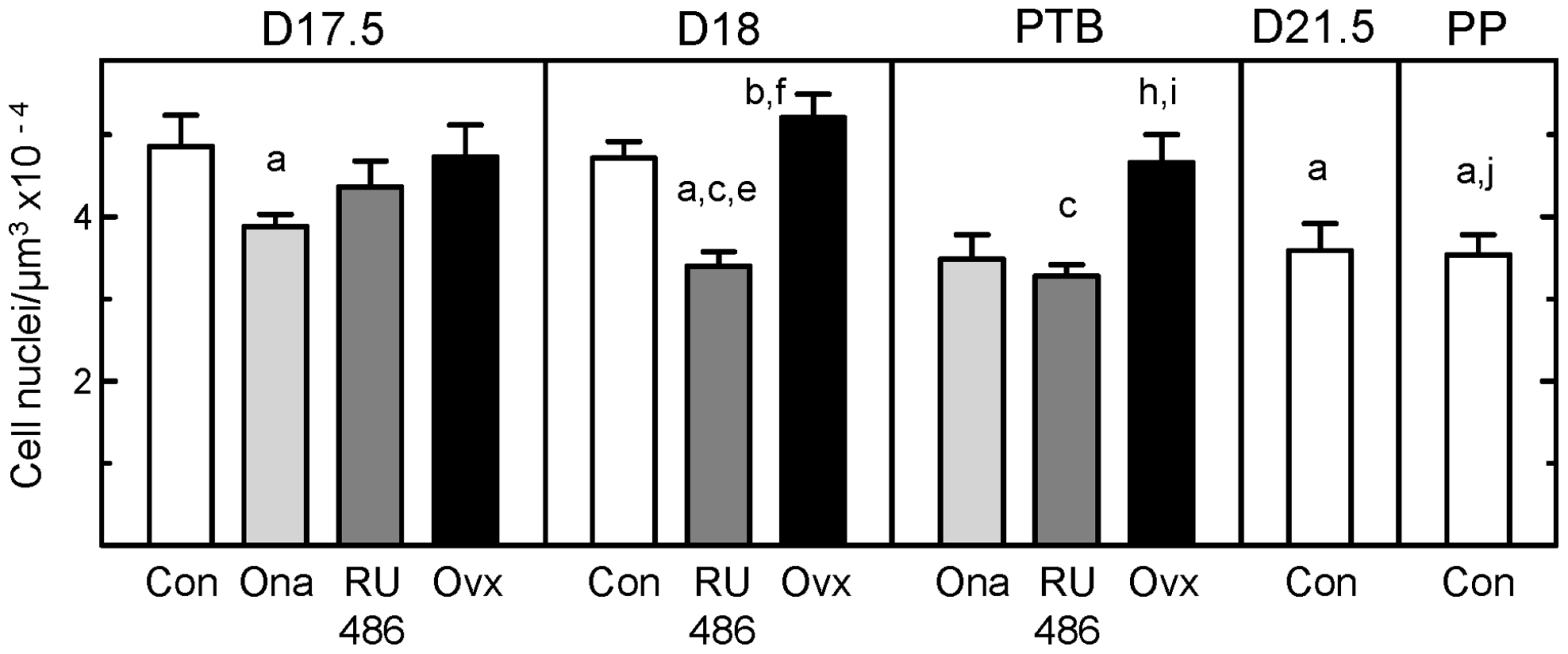
Reduced cell density in the cervix with preterm and term birth. Number of cell nuclei/area in sections of cervix (mean±SE, n = 5–7 rats/group at each postbreeding date; 6 sections each with 6–8 non-overlapping photomicrographs/rat, i.e., average of 36–48 photomicrographs/rat) from groups of rats relative to days postbreeding or postpartum (PP) as described in Figure 1 legend. Decreased cell nuclei/area indicates hypertrophy and edema of tissue. For perspective, CN density for all groups were significantly reduced compared to NP = nonpregnant controls (9.3±0.45 µm^3^ ×10^−4^, n = 6 [30]; p<0.05 ANOVA). A Student's t-test compared data for Con versus Ona groups day 17.5 postbreeding (p = 0.04, t = 2.4, df = 8). Letters denote statistical significance from groups assigned letters ^a^ Con D17.5 through ^k^ Con D21.5. By example, ^f^ over the Ovx D18 group indicates a significant increase in cell nuclei number/area compared to that in RU486 D18 while the absence of letters ^d or k^ indicate the lack of significant difference for any group in comparison to Ovx D17.5 or Con D21.5 groups, respectively.

Based upon analyses of subepithelial and deeper stromal areas (excluding blood vessels, luminal epithelium, and lumen), the number of cell nuclei per area of cervix in pregnant controls declined by the day before birth relative to that earlier in pregnancy (Figure 3, p<0.05 Con D21.5 vs D17.5). Within 8 h of Ona treatment (about 12 h before birth), fewer cell nuclei per area were found in the cervix from rats on day 17.5 postbreeding versus D17.5 controls. RU486-treated rats also had fewer cell nuclei (about 12 h before birth), with a significant decline by day 18 postbreeding compared to that in the Con D17.5 and D18 groups. Therefore, the prepartum cervix of rats given PR antagonists had prematurely hypertrophied by 8–24 h to the same density as found in D21.5 controls at term. By contrast, removal of the ovaries did not affect the density of cells in the cervix (p>0.05 Ovx D17.5 vs Con D17.5). Rather, cell densities in the prepartum and postpartum cervix of Ovx rats (D18 and PTB groups) were greater compared to that in prepartum Ona D17.5 and RU486 D18 groups, as well as Con D21.5 mice. Thus, the ovariectomy-induced decline in systemic progesterone and preterm birth did not further hypertrophy the cervix compared to that found in peripartum controls at term.

### Census of macrophages in the cervix with preterm birth

**Figure 4:**
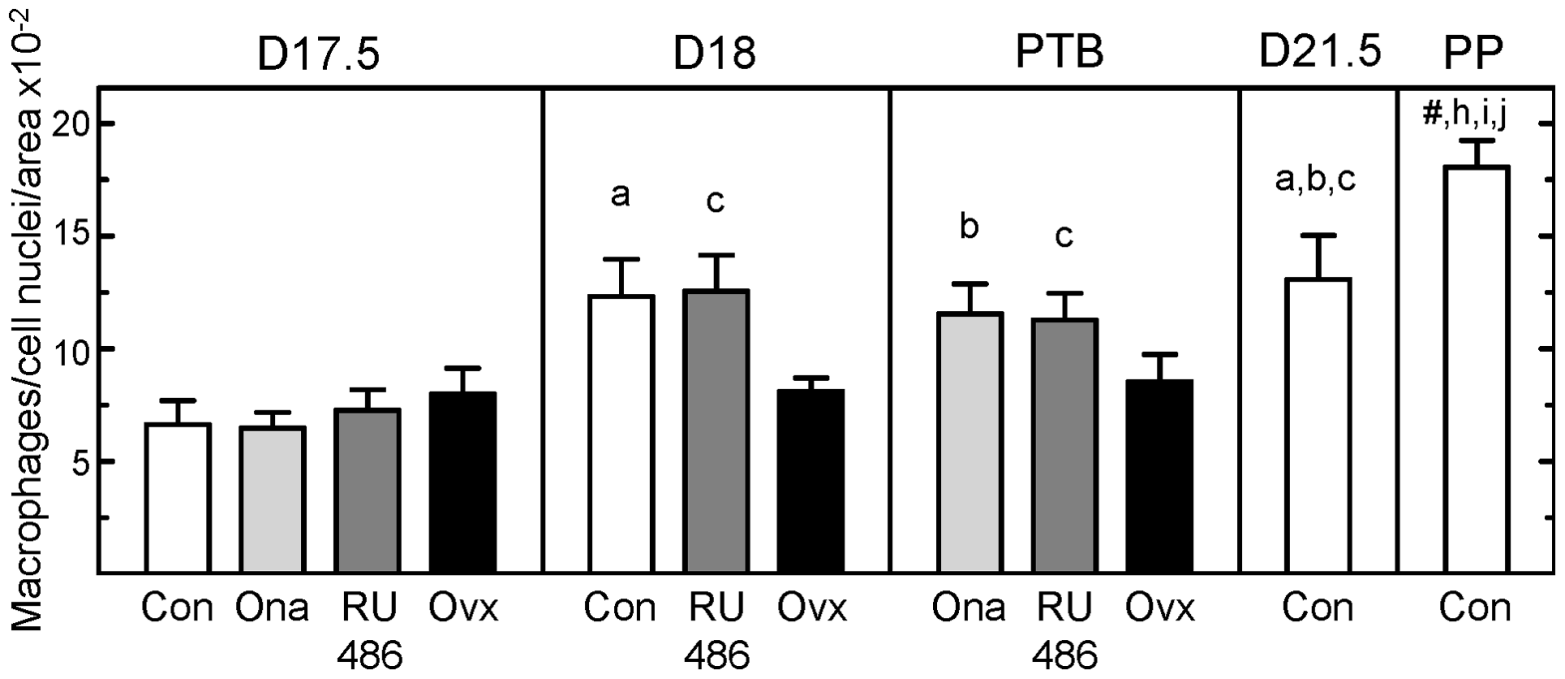
Increased density of macrophages in peripartum cervix at term and with PR antagonist-induced preterm birth. Macrophages normalized to cell nuclei/area to account for tissue hypertrophy in sections of cervix (mean±SE, n = 4–7 rats/group at each postbreeding date). Groups and statistical comparisons are the same as in legends to Figures 1 and 3 (p<0.05 ANOVA; Con D21.5 vs D17.5, Student's t-test p = 0.04, t = 2.5, df = 8). ^#^ p<0.05 versus Con D17.5, D18, and D21.5 groups. For perspective, all pregnant rats in the present study had significantly more macrophage in cervix sections compared to that in nonpregnant rats (0.41±0.102/µm^3^ x 10^−2^, n = 6 [30]; p<0.05 ANOVA).

Within stromal and subepithelial regions, macrophages in the cervix of controls increased as pregnancy progressed to term in controls and in PR antagonist-treated rats with preterm birth (Figure 4). The density of macrophages in the cervix was greater in Con D18 and prepartum Con D21.5 groups verses Con D17.5 rats (p<0.05 ANOVA). Similarly, more macrophages were found in sections of cervix from RU486 D18, RU486 PTB, and Ona PTB groups compared to that in prepartum D17.5 rats given Ona or RU486, respectively. The postpartum increase in macrophages in the cervix of controls at term was greater than found in postpartum Ona- and RU486-treated rats with preterm birth. By contrast, the number of macrophages in the cervix did not change within 8h of ovariectomy (p>0.05 Ovx D17.5 vs Con D17.5). In addition, fewer macrophages were present in the postpartum cervix of Ovx PTB rats compared to that in controls (p<0.05 ANOVA). Thus, a stable census of macrophages was maintained as the remodeling process in the cervix concluded in ovariectomized rats with preterm birth.

**Figure 5:**
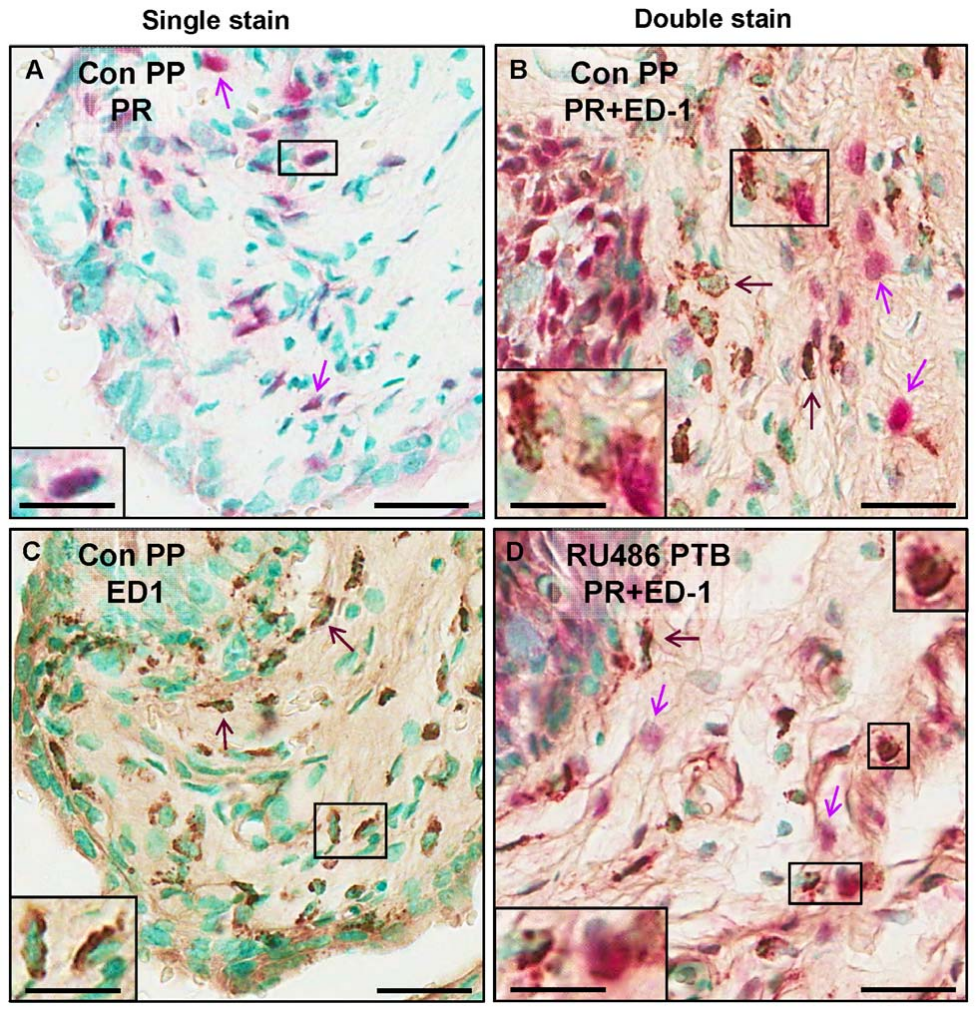
Photomicrographs of single (PR or ED1) and double-stained cells in sections of cervix from rats at term and with PR antagonist-induced preterm birth (RU486 PTB, day 18 postbreeding). **A, B**. PR or ED1 stained section, Vector Red by alkaline phosphatase or brown HRP-DAB respectively, from a Con PP rat on day of term birth (day 22 postbreeding). **C, D**. PR- and ED1-stained cervix sections from a postpartum Con PP rat at term or RU486 PTB rat with preterm birth. Cell nuclei were visualized with a methyl green counterstain. These photomicrographs are representative of observations from analyses of cervix sections from 3–4 rats/group with available sections (Con D17.5, 18, PP; RU486 D17.5, PTB; Ona D17.5; Ovx D18. Double-stained PR and ED1 cells were a sparse minority of the total number of single PR or ED1 stained cells. Staining characteristics, including the predominance of single-stained cells, are consistent with a previous report in the cervix from postpartum women [32]. Arrows indicate examples of single stained cells. Examples of putative PR+ED1-stained cells are highlighted in the box in panels B and D (magnified inset). Scale bars indicating 15 µm and 30 µm (inset), respectively. See Methods for details.

To determine whether antiprogestin actions are directly mediated by macrophages, sections of cervix from rats in every group were processes to visualize macrophages and cells that contain progesterone receptors. Using Vector red, PR stained cells were found throughout the cervix from rats in all groups controls irrespective of day of pregnancy, treatment, or timing of delivery (Figure 5A). Dense pink-red deposits covering the entire cell nucleus and often obscured the nuclear methyl green counterstain (purple arrows). Subregions with red-stained PR cells included areas where brown-stained macrophages typically reside (brown arrows) in the subepithelium and stroma (Figure 5B), as well as other locations including the epithelium adjacent to the lumen. In sections double stained for PR and ED1 Figure 5 C, D), cells were predominantly red or brown, i.e., distinct separate populations of PR or macrophage cells. A sparse minority of cells in the cervix, irrespective of treatment group, appeared to have both red and brown stain (inset highlighted area in box). Many of these apparent double-stained cells had a blush of red stain that was characteristic of PR with dark spots that were typical of an ED1-stained adjacent to or surrounding the methyl green counterstained cell nucleus. Photomicrographs of sections from prepartum and postpartum controls and rat given RU486 that gave birth preterm were typical of the quality and distribution of single and double stained cells in all groups including Ona-treated and Ovx rats. These findings suggest that the macrophages with PR were a sparse minority of the total population of cells that were exclusively PR or macrophages lacking PR.

**Figure 6:**
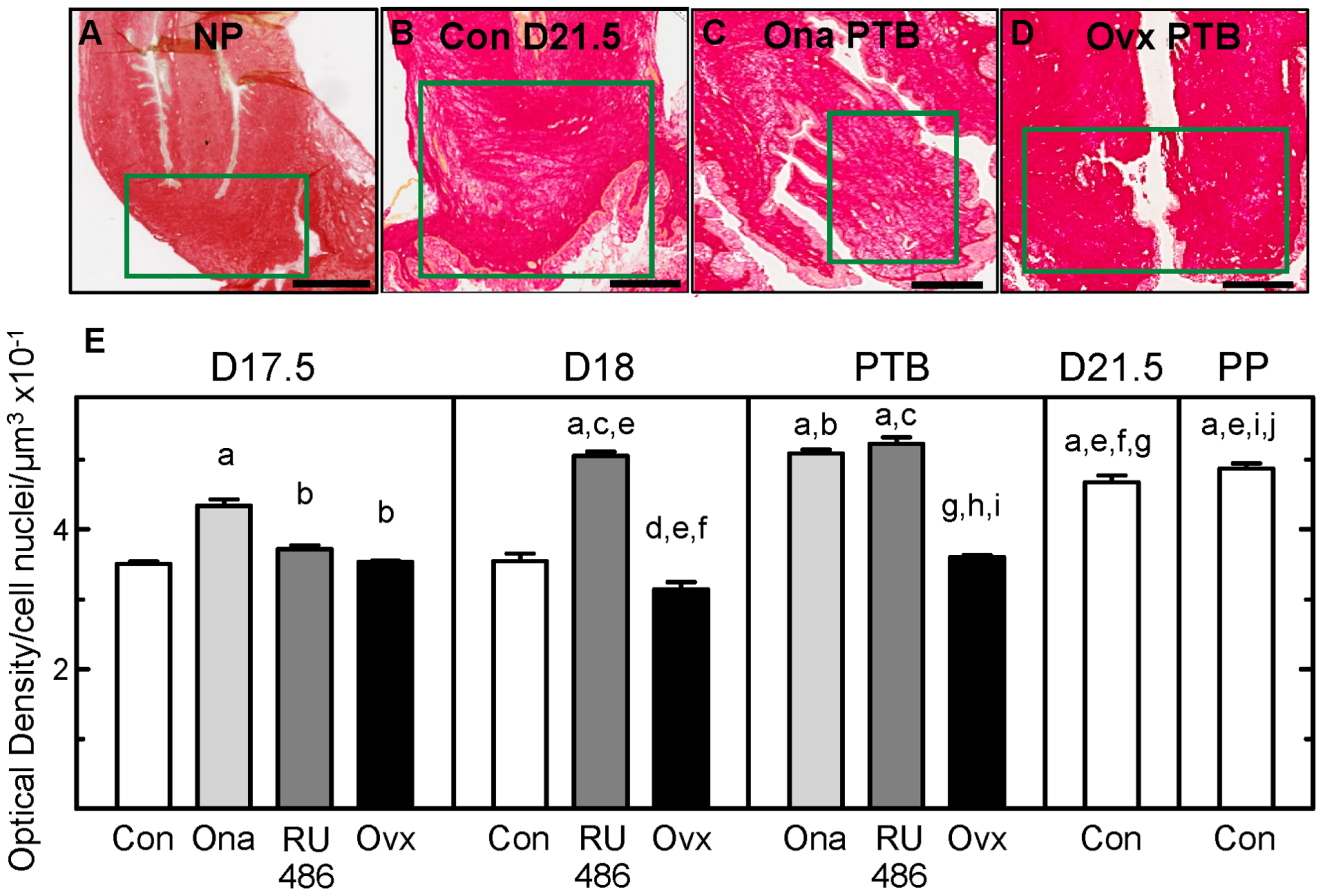
Degradation of collagen content and structure in the peripartum cervix at term and in preterm birth. Photomicrographs of picrosirius red dye-stained cervix sections from a rat that was **A**. nonpregnant (NP; [30]), or from rats in **B**. Con D21.5 preterm day 21.5 postbreeding, **C**. Ona PTB or **D**. Ovx PTB groups. Intensity of orange-red stain reflects extracellular collagen content and cross-linked fiber structure. Photomicrographs were obtained at magnifications of 40x, stitched, and sized to size. Scale bars indicate 1 mm. **E**. Optical density (mean±SE, n = 5–7 rats/group at each postbreeding date; analyses of 27 non-overlapping sections/rat) of picrosirius red-stained sections of cervix from Con, PR antagonist-treated, and Ovx rats normalized to cell nuclei number/area in each individual to account for tissue hypertrophy and edema. SE bars on some groups are small and obscured by the mean. Optical density is inversely related to collagen content and structure (details in Materials and Methods). Group designations and statistical comparisons are the same as in Figure 1 legend. By example, the ^b^ assigned to the Ona D17.5 group indicates, by comparison, a significant decrease in OD of cervix sections from the RU486 D17.5, Ovx D17.5, and Ona PTB groups (greater collagen content and structure). By contrast, lack of a letter ^k^ indicates not significant differences in comparison to the Con D21.5 group. For perspective, the OD of cervix sections from all groups of pregnant rats were significantly increased, i.e., reduced collagen content and structure, compared to that in nonpregnant controls (1.506±0.0309 µm^3^ ×10^−1^, n = 6 [30]; p<0.05 ANOVA).

Within the extracellular matrix of the cervix, collagen content and structure declined before term and, with treatment, before preterm birth. The overall transition from an unremodeled cervix to a prepartum remodeled cervix is visualized by the dark orange-red dye in a section of cervix of a nonpregnant rat, an indication of dense, structured extracellular collagen, compared to the light, relatively sparse stain in a prepartum cervical section from a rat on day 21 postbreeding (Figure 6 A–D). Picrosirius red-stain was reduced in sections from controls by D21.5, the afternoon of the day before birth, and from the Ona PTB group compared to that from NP [30] or Ovx PTB rats. Replicating previous findings, the optical density of picrosirius-stained sections increased before term birth in controls (Figure 6 E; p<0.05 Con D21.5 vs ConD17.5). PR antagonist treatments similarly increased the optical density of prepartum cervical sections compared to that in controls (p<0.05 Ona D17.5 vs Con D17.5, RU486 D18 versus Con D18). Thus, PR antagonists reduced collagen content and structure in advance of preterm birth to an extent comparable to that in the prepartum cervix of controls at term. The optical density in the postpartum cervix of PR antagonist-treated rats similarly increased as in controls compared to that in D17.5 groups, respectively. By contrast, the optical density of sections from the cervix of Ovx rats initially was unchanged (p>0.05 Ovx D17.5 vs Con D17.5) or reduced compared to that in controls on the same day postbreeding or the prepartum D21.5 group (p<0.05 Ovx D18 vs Con D17.5, Con D18, RU486 D18, and Ovx D17.5). The collagen content and structure of the prepartum cervix in Ovx D18 rats increased with preterm birth the next day (p<0.05 Ovx PTB vs Ovx D19).

## Discussion

The present study addressed the hypothesis that PR antagonists advance critical characteristics of cervical remodeling that occur at term. Though serum progesterone concentrations remain elevated at least 8h after PR antagonist treatment, cell and collagen densities per area of cervix were reduced – an indication that hypertrophy, edema, and degradation of the extracellular matrix had occurred in the cervix before preterm birth. Moreover, enhanced density of macrophages in the cervix with PR antagonist treatment relative to controls on the same day postbreeding, preterm for RU486 or for Ona by the day of preterm birth, further suggests an accelerated remodeling process. These findings are the first morphometric evidence that antagonism of PR-mediated actions advance inflammatory processes in the cervix in association with preterm birth that are similar to remodeling characteristics that occur by the day before birth at term. In addition, the results in controls are the first replication of previous findings in another rat strain and corroborate findings in several strains of mice [4], [30]. Collectively the evidence indicates that structural remodeling of the cervix at term coincides with an increased presence of macrophages relative to that earlier in pregnancy. These findings promote and extend the important concept suggested by Mesiano et al and others [33]–[36] that loss of PR-mediated actions remodels the cervix at term in this model of preterm birth.

The present study is also the first to use a systematic approach to evaluate cellular and structural remodeling by the cervix to PR antagonist treatment. Assessment of a sufficient area of cervical stroma and subepithelium permitted quantitative comparisons of remodeling characteristics among various groups in the study. Thus, the present study in rats clarifies and extends initial indications from previous reports in mice and women [26], [37]. Reduced density of cell nuclei per area reflects extracellular remodeling in the cervix, a result from cellular hypertrophy, as well as edema to loosen the network of collagen fibers that contribute to changes in biomechanical tensile strength as pregnancy progresses to term. The decline in content and structure of collagen in stroma and subepithelium in controls and after PR antagonist treatment in the extracellular locale that is likely to regulate macrophage census and activity further supports a role for PR in a final common mechanism for degradation of collagen in the remodeling processes at term and with this etiology of preterm birth. Moreover, the increased presence of macrophages in the cervix after treatment of pregnant rats with a pure PR antagonist is a novel addition to the literature. The comparable effects on remodeling after treatment with Onapristone, a pure progesterone receptor antagonist, or RU486, a mixed progesterone and glucocorticoid receptor antagonist, indicates that antagonism of the classic nuclear progesterone receptor, but not necessarily the glucocorticoid receptor, is a critical part of the mechanism that promotes premature remodeling of the cervix. Furthermore, the loss of actions by progesterone on cells in the cervix may not directly involve macrophages given that few stain for PR, a finding consistent with those in a previous report in the cervix from postpartum women [32].

A second major novel finding of this study was that characteristics of the inflammatory-associated remodeling process at term and after PR antagonist treatment-induced preterm birth did not occur after ovariectomy. Removal of the ovaries did not promote hypertrophy, extracellular collagen degradation, or recruitment of macrophages in the cervix during the period leading up to preterm birth. Absence of these remodeling characteristics coincided with dystocia in some Ovx rats, a possible indication that an increased presence of macrophages and further degradation of extracellular collagen are important to complete the ripening process. The precipitous decline in systemic progesterone concentrations in Ovx rats in this study parallels evidence in ovariectomized pregnant mice in which preterm birth results without structural remodeling of the cervix after treatment with progesterone ceases, but estradiol concentrations are sustained [38]. Whether estradiol in circulation affects immigration of immune cells is not indicated by findings that a stable population of macrophages resides in the uterus throughout a physiological range of serum estradiol during the estrus cycle in mice [39]. Also similar to the present study, RU486 treatment of pregnant rats induced preterm birth within 28 h as serum estradiol increased 2-fold [40]. The possibility remains that estradiol may act synergistically to facilitate the remodeling process [41] or antagonize progesterone actions [42]. However, differences in preterm remodeling characteristics between Ovx and other groups in this study direct attention to the possibility that the cervix maybe sufficiently remodeled in the rat by 3 or more days before term when serum progesterone is elevated and comparable to near peak concentrations in previous studies [7], [9], [14], [43]. The important role of relaxin in remodeling the cervix during this period of pregnancy has been recognized [44]. By contrast, development of a contractile phenotype by uterine smooth muscle is coincident with labor and the decline in progesterone in circulation in rodents. These and subsequent findings support the understanding, first conceived by Csapo, that withdrawal of progesterone promotes a cascade of processes that initiate labor [45]. Clearly, systemic progesterone withdrawal does not drive characteristics of cervical remodeling associated with birth at term or with PR antagonist-induced preterm birth. Rather in the final common mechanism for cervical remodeling, blockade of PR-mediated actions both at term and with some instances of preterm birth as indicated in the present study, along with enhanced steroid metabolism may contribute to a local withdrawal of progestational actions in the prepartum cervix [14]. Thus, rodents may be more similar to higher vertebrate species in that systemic progesterone concentrations are sustained during the period associated with remodeling of the cervix in preparation for birth.

In a broader context, the rise in serum progesterone after PR antagonist treatments indicates that blockade of central nervous system PR abrogates negative feedback control to drive gonadotropin hormone secretion and ovarian steroidogenesis [21]. Evidence that PR antagonists act on negative feedback centers in the brain [46], [47] contributes to the novel consideration that parasympathetic-mediated central innervation [6], in addition to local progesterone actions, may recruit macrophages and promote inflammatory processes that remodel the cervix prior to birth at term. Whether specific parasympathetic neuromediators affect characteristics associated with cervical remodeling remains to be determined. Another approach of potential clinical relevance to regulate aspects of the cervical remodeling process is the use of progesterone prophylaxis for prevention of preterm birth in patients at high risk for preterm birth [48]. Considerations for therapeutic efficacy to arrest premature cervical remodeling may depend upon optimal dosing, route of administration of progesterone, clinical indications, and previous history of preterm birth [49], [50].

In summary, the present findings suggest that loss of nuclear progesterone receptor actions promote cervical remodeling and parturition both at term and with preterm birth. Antagonism of classic nuclear progesterone receptors advances critical cellular characteristics of remodeling including i) hypertrophy (decreased cell nuclei density), ii) degradation of the extracellular collagen matrix (increased optical density), and iii) inflammatory processes (increased recruitment of macrophages), in the cervix that are found to occur at term. However, not all etiologies for preterm birth are the same because evidence in Ovx groups suggests that structural characteristics of the cervix may be sufficiently compliant well before term. Resident macrophages may await signals, either from an indirect loss of progesterone receptor-mediated actions or a central parasympathetic cue, as part of a final common mechanism for remodeling of the cervix at term across species. Further studies of progesterone supplementation or neuroimmune treatments are needed to determine if a therapeutic regimen may optimally forestall local inflammatory processes in the cervix of women at risk for preterm remodeling of the cervix and, ultimately, improve neonatal outcome.
